# Influence of ZnO and TiO_2_ additions on structural, physical, and electrical properties of LaCo_0.3_Mn_0.7_O_3_ perovskite

**DOI:** 10.1038/s41598-025-11040-8

**Published:** 2025-07-19

**Authors:** M. A. Hessien, H. E. H. Sadek, H. H. Abo-Almaged, M. A. Taha, R. M. Khattab

**Affiliations:** 1 Refractories, Ceramics and Building Materials Department, National Research Centre, Dokki, Egypt; 2 Solid-State Physics Department, National Research Centre, Dokki, 12622 Cairo Egypt; 3 Pharos University, Canal El Mahmoudia Street, Smouha, Alexandria, Egypt

**Keywords:** Perovskite, Microwave-hydrothermal method, Mechanical, Electrical, Magnetic properties, Nanoscale materials, Structural materials

## Abstract

Lanthanum manganite-lanthanum cobaltite (LaCo_0.3_Mn_0.7_O_3_) solid solutions have been investigated as potential candidates for ceramics, catalysts, and sensors. A microwave hydrothermal process was used to prepare LaCo_0.3_Mn_0.7_O_3_ powders, fired at temperatures of 600, 1100, and 1300 °C. The effect of different transition metal additions (ZnO and TiO_2_) on the optimum selected perovskite was studied. X-ray diffraction (XRD), scanning electron microscopy (SEM), X-ray photoelectron spectroscopy (XPS), bulk density, apparent porosity, and electrical, mechanical, and magnetic properties were used to characterize the obtained materials. The results indicated that the optimum perovskite phase was obtained at 1100 °C. Different amounts of ZnO and TiO_2_ did not react with the perovskite, which was a separate phase. The maximum density of about 4.55 g/cm^3^ was observed for the sample containing 10 wt% of TiO_2_, and a minimum porosity of about 2.5% is obtained for the sample containing 20 wt/% of TiO_2_, While the bulk density reached 5.7 g/cm^3^ and porosity of about 2% after the addition of 30 wt% of ZnO. ZnO enhances the sinterability, hardness, and conductivity of perovskite structure. On the contrary, TiO_2_ was added up to 20 wt% enhances hardness properties, while the conductivity is decreased by TiO_2_ addition. The hardness value reaches 6.76 in the case of 20 wt% TiO_2_ and 5.6 in case of 30 wt% ZnO addition. This is due to the formation of a large amount of the liquid phase, which acts as a barrier to charge transfer. The electrical properties of the LaCo_0.3_Mn_0.7_O_3_ matrix with varying TiO_2_ and ZnO contents, sintered at 1100 °C, were investigated through conductivity and resistivity measurements at 28 °C, 100 °C, 300 °C, and 500 °C. Upon increasing the TiO_2_ content from 10 to 30 wt% resulted in increased resistivity from 1.5 × 10^8^ to 3.3 × 10^8^ Ω cm and decreased conductivity from 6.5 × 10^–9^ to 3.5 × 10^–9^ S/cm. Conversely, increasing the ZnO content increased the conductivity from 3.5 × 10^–9^ to 6.3 × 10^–9^ S/cm and decreased the resistance from 2.85 × 10^8^ to 1.5 × 10^8^ Ω cm. In addition, all samples exhibited paramagnetic properties that decreased with the addition of transition metals.

## Introduction

Perovskite-type oxides are inorganic materials with the formula ABO_3_, where A is a rare/alkaline earth metal cation, and B is a transition metal ion^1^. These materials have attracted interest due to their characteristics, including controllable electrical conductivity and ferromagnetic, antiferromagnetic, or colossal magneto-resistance properties. Perovskites are used in biomedicine, sensors, refrigeration, magnetic memory, and catalysis^2–7^. The perovskite structure is usually cubic, though phase transition can occur at low temperatures, resulting in various magnetic structures^8^. The perovskite lattice accommodates ions of different sizes and charges, where changes in A- and/or B-site ions or stoichiometry affects electrical properties. This is due to their atomic structure and electronic energy bands, making them subjects of basic and applied research^9–11^.

Perovskite oxides are fabricated using ceramic techniques under ambient air pressure. The process involves mixing stoichiometric proportions of solid precursor oxide reactants, followed by heating at high temperatures (usually several hours at 1000 °C). The grinding and heating cycles continue until achieving the pure phase. While this synthesis method under thermodynamic equilibrium conditions is versatile, it has disadvantages including high temperatures, volatile reactants, repeated grinding, and high energy consumption^12^. This necessitates alternative methods to produce homogeneous thin perovskite oxides, such as co-precipitation, sol–gel processes, and solution combustion techniques.

Most methods use organic polymers as complexing agents, including Pechini-type complexes, polyesters, polyethylene glycol (PEG), and polyacrylic acid (PAA). Solution combustion synthesis offers easy processing, simple setup, and energy efficiency. It involves mixing ingredients with a fuel (urea or citric acid) and oxidizer (nitrates) in an aqueous medium. Microwave heating differs from traditional heating by generating heat inside the sample through microwave interaction^13^. This enables uniform heating of the sample volume, improving the product’s properties and homogeneity. Various metal oxides, including spinels, Cr_2_O_3_, Fe_2_O_3_, and (La,Sr,Ca)_X_O_3_ perovskites, have been synthesized using microwave heating. Microwaves are now used to create oxide nanoparticles under hydrothermal conditions^14^. Perovskite powder can be prepared hydrothermally in one step without expensive reagents^15^.

LaMnO3 is a perovskite with an ABO3 structure in oxide fuel cells, compatible with yttria-stabilized zirconia electrolyte as cathode material in solid oxide fuel cells^6^. LaMnO3 thin films can be prepared using methods like pulsed electron deposition, sol–gel, and microwave combustion^2,16,17^. Researchers described stoichiometric and nonstoichiometric LaMnO3 solid-state synthesis. The samples were prepared using mechanochemical activation^18–23^. Chemical co-precipitation was used in LaMnO3 production. LaMnO3 was prepared using sol–gel technique with varying-sized particles^24^. The hydrothermal technique for synthesis is detailed in^25^. Microwave plasma can produce LaMnO3^26^. Glycine-nitrate and spray drying yield nonstoichiometric samples^27,28^. The citrate pattern (Pechini method) was used^29–31^ for nonstoichiometric samples. The Pechini method’s synthesis outcomes are detailed in^26,32,33^. The metals Fe, Co, Ni, and Mn on the B-site show high activity for oxygen evolution reaction (OER) at anode and oxygen reduction reaction (ORR) at cathode^34,35^. Partial substitution of a secondary transition metal for B-site cation boosts electrocatalytic activity through synergistic effects. The compounds’ porous structure encourages oxygen or electrolyte migration into perovskite materials^36–39^.

Kondakiddi et al.^40^ states that substituting A-ion or B-ion with different metals alters valence states, enhancing oxygen movement within the crystal. Manganese oxides show strong redox properties, and manganese’s higher valence states facilitate oxygen mobility. Palcut et al.^41^ found LaMnO_3_ has higher cation mobility than LaCoO_3_, suggesting Mn replacement with Co could reduce mobility when slower diffusion is needed^41^. LaMnO_3_’s defect chemistry due to Mn’s stability in higher oxidation states indicates Co substitution could alter the material’s defect chemistry and properties^41^. In cobalt ferrite systems, Mn substitution affects magnetic properties, enhancing magnetization in certain compositions^42^, due to differences in ionic radii and valence states between Mn and host cations^42,43^.

The sinterability of doped perovskite can be improved by adding transition metal oxides like ZnO, CoO, NiO, CuO, and TiO_2_^44–49^. According to Wang et al*.,* ZnO increased.

BaCe_0.5_Zr _0.3_Y_0.2_O_3−δ_’s relative density to over 97%, with its dense body showing 1.35 × 10^−2^ S/cm electrical conductivity at 600 °C^45^. The BaO–ZnO eutectic stimulated the perovskite through liquid-phase sintering, supported by phase diagrams. They noted excess ZnO decreased perovskite’s electrical conductivity. Limited information exists on the optimal ZnO concentration for enhancing perovskite’s sinterability and electrical conductivity. According to Amsif et al. Zn^2+^ can integrate into the perovskite lattice due to similar ionic radii between Zn^2+^ (0.74 Å) and Zr^4+^ (0.72 Å)^50^. Zn^2+^ ions incorporated into the perovskite lattice create oxygen vacancies and B-site defects to maintain electrical neutrality. Oxygen vacancies and B-site flaws encourage mass transport, explaining ZnO’s improvement of perovskite’s sinterability^50,51^. However, no studies have examined Zn incorporation into perovskite using theoretical or practical techniques. Understanding the sintering mechanism of ZnO-added perovskite is essential to comprehend the material’s properties. These findings help improve the reliability of perovskite in proton-conducting ceramic-electrolyte fuel cells (PCFCs), as ZnO-added perovskite’s characteristics are crucial for material properties^44^.

LaMn_0.3_Co_0.7_O_3_ is a perovskite oxide material that has potential applications in various energy-related fields, particularly in solid oxide fuel cells (SOFCs) and electrochemical devices. In SOFCs, LaMn_0.3_Co_0.7_O_3_ can be used as an electrode material due to its favorable properties. Lanthanum-based perovskites like La_0.75_Sr_0.25_Cr_0.5_Mn_0.5_O_3_ have been studied as electrode materials for SOFCs, showing high electrochemical activity for oxygen reduction and hydrogen oxidation reactions^52^. The partial substitution of manganese with cobalt in LaMn_0.3_Co_0.7_O_3_ may further enhance its catalytic properties and electrical conductivity, making it suitable for SOFC applications. Interestingly, the properties of such materials can be tuned by varying the composition and synthesis methods. For instance, strontium substitution in lanthanum cobaltite (La_1-x_Sr_x_CoO_3_) has been shown to improve electrical conductivity^53^.

The results of a study on the impact of adding Co ions to LaMno_3_ to create La Co_0.3_Mn_0.7_O_3_ using a microwave hydrothermal technique are presented herein. Additionally, research has been conducted on how various transition metals, such as TiO_2_ and ZnO, affect the process of creating La Co_0.3_Mn_0.7_O_3_ materials. In addition, several investigations have been conducted on the acquired materials to ascertain their phase composition, structure, and physical, mechanical, electrical, and magnetic properties to produce a product that can be used in various applications, including ceramic fields, catalysts, and sensors.

## Experimental procedure and characterizations

### Chemicals

All chemicals were purchased from Sigma Aldrich and used as supplied. These were La(NO_3_)_3_·6H_2_O (99%), CoCl_2_·4H_2_O (99.9%), MnCl_2_·4H_2_O (99%), ZnO, TiO_2_ andNH_4_OH.

### Experimental procedure

A laboratory-built microwave-heating-autoclave system with a Teflon polytetrafluoroethylene (PTFE) inner vessel that operates at 2.45 GHz and can reach a controlled temperature of up to 220 °C was used to perform the microwave treatment. The system was equipped with an auxiliary cooling/heating device that allowed it to function at a fixed temperature for an extended amount of time while retaining the input power of the microwave radiation during the reaction period.

**Fig. 1:**
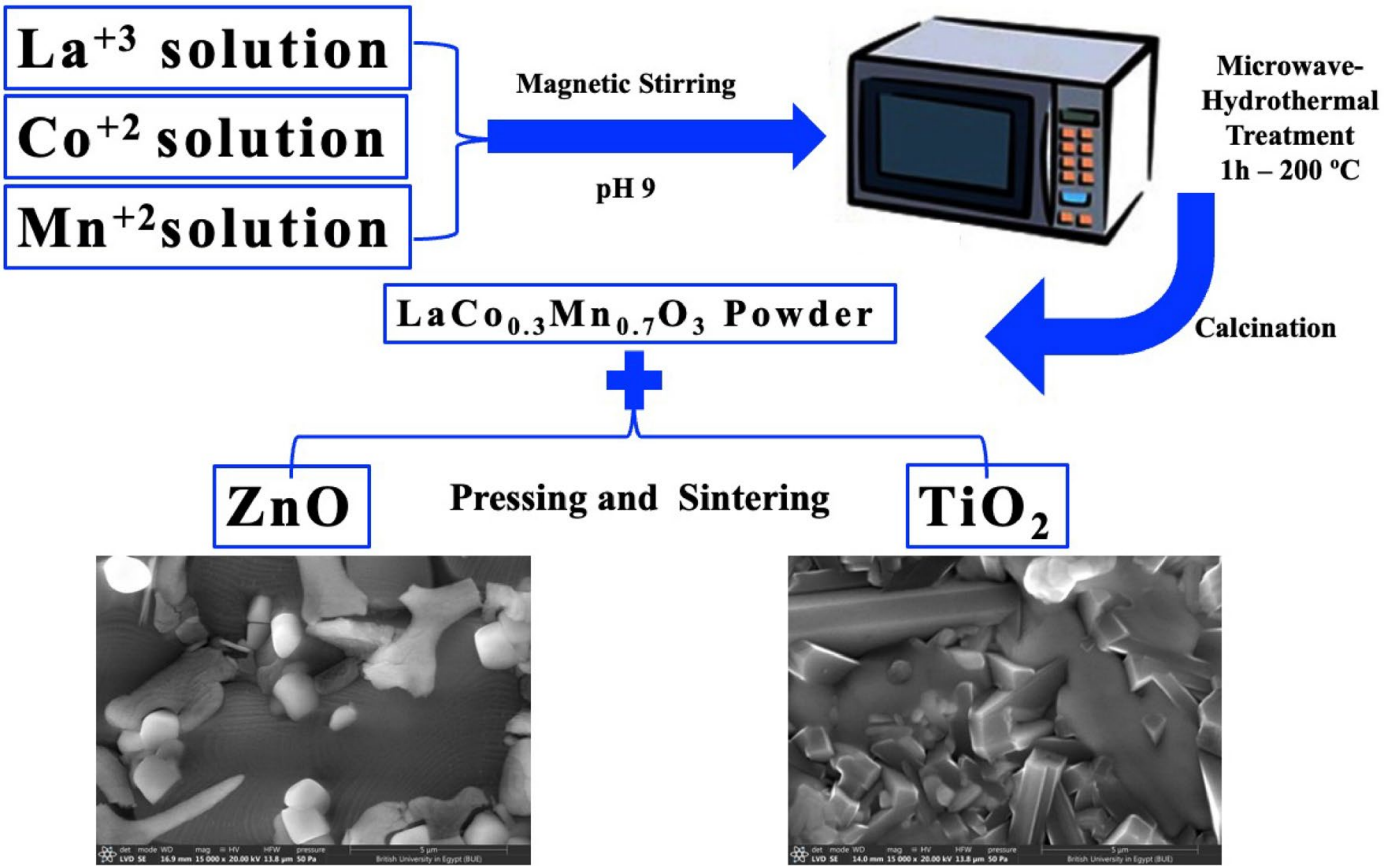
A schematic representation of the experimental method.

To prepare LaCo_0.3_Mn_0.7_O_3_ (named PP), Stoichiometric amounts of lanthanum salt, cobalt salt, and manganese salt were weighed and dissolved in distilled water to prepare 0.2 M solution. Ammonia solution was added dropwise to the salt solution while under magnetic stirring until pH 9 was reached. After the solution was moved into a sealed PTFE autoclave, the system underwent an hour of heat treatment at 200 °C. The powders that were left over were centrifuged, cleaned in distilled water, and oven-dried for twenty-four hours at 85 °C. The resulting powder was named (PP) and was burned at 600 °C, 1100 °C, and 1300 °C until perovskite formation was optimized. ZnO and TiO_2_ (10, 20, and 30 wt%) were added in varying amounts to the powder with the best perovskite crystallization. The powder mixture was sintered at 1100 °C after pressing (50 MPa). The bodies prepared with 10, 20, and 30 wt% of TiO_2_were named T10, T20, and T30. The bodies prepared with 10, 20, and 30 wt% of ZnO were named Z10, Z20, and Z30. A schematic representation of the experimental method is shown in Fig. 1.

### Characterizations

The phase composition of powder and bodies was examined using the X-ray diffraction (XRD) technique with monochromatic Cu Ka radiation (D 500, Siemens, Mannheim, Germany) and a scanning rate of 2 deg./min. The crystallite size was determined by Scherrer Eq. (1).

1 $$D = 0.9\lambda /B\;\cos \theta$$

X-ray photoelectron spectroscopy (XPS) was utilized to investigate the surface composition of the samples. This analysis was conducted using a VG-Microtech Multilab 3000 system (Thermo-Scientific, Waltham, MA, USA), which incorporates an Al Kα radiation source with an energy of 1253.6 eV.

The fired powder and bodies were examined under a scanning electron microscope (SEM) with FEI, QUANTA FEG, 250. Archimedes’ method was used to calculate the specimens’ bulk density and apparent porosity. Vickers microhardness (H_v_) was evaluated in ambient laboratory circumstances with a continuous indenter dwell time of 10 s using a Shimadzu-HMV (Japan) microhardness tester with a 100 g load. Equation 2 was used to compute Vickers microhardness^54^.

2 $$H_{v} = 1.854P/D^{2}$$

P is the applied indentation load, and D is the measured diagonal.

Electrical conductivity and resistivity were measured at different temperatures using a Keithley 6517B electrometer as described in^55^. It is crucial to remember that five measurements of the bulk density, apparent porosity, microhardness, and electrical characteristics were performed to guarantee the accuracy of the results. A Lakeshore VSM 7410 model with a 3 T magnet was used for the magnetic measurements (M versus H).

## Results and discussion

### Phase compositions

**Fig. 2:**
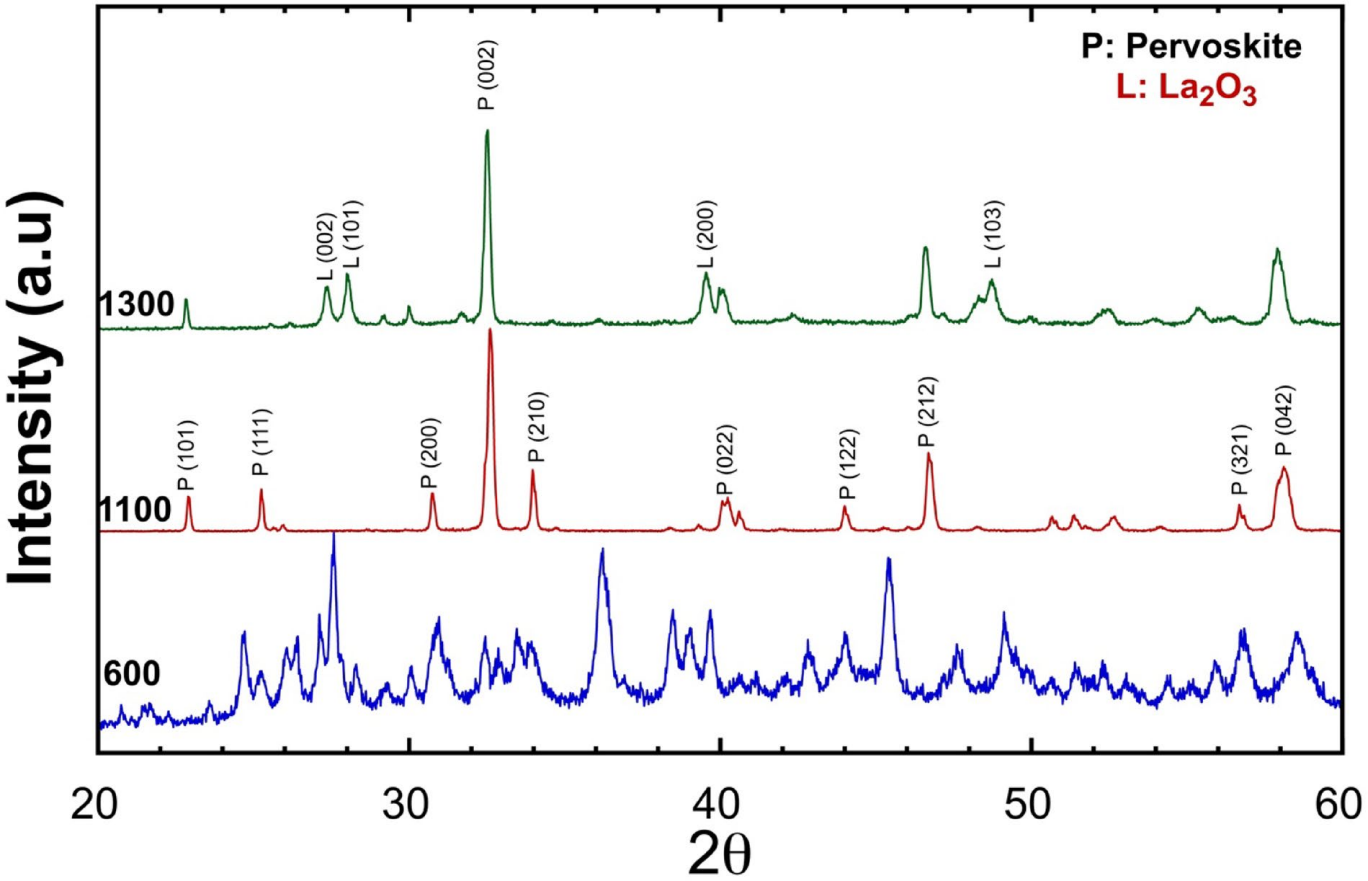
XRD patterns of LaCo_0.3_Mn_0.7_O_3_ powder calcined at different temperatures 600 °C, 1100 °C, and 1300 °C.

The XRD pattern in Fig. 2 was employed to determine the phase makeup of Sample PP after subjecting it to heat treatment at 600 °C, 1100 °C, and 1300 °C. The sample displayed broad and wide peaks when the calcination temperature was 600 °C. Nevertheless, as the calcination temperature increased to 1100 °C, the peaks became more pronounced and sharper, indicating that the powder had a crystalline structure. The peaks corresponding to the (101), (111), (200), (002), (210), (022), (122), (212), (321), and (042) planes of the perovskite orthorhombic structure were identified at 22.81°, 25.28°, 30.74°, 32.59°, 33.99°, 40.15°, 43.94°, 47.76°, 56.7°, and 58.1°. Perovskite crystallization can take place at temperatures as high as 1100 °C. The crystals exhibit an orthorhombic structure with the Pnma (62) space group^56^, as demonstrated by detecting PDF#Card number89-0682 at 1000 °C in sanitary LaMnO_3_. This orthorhombic structural quality was also evident in the LaCo_0.3_Mn_0.7_O_3_ samples sintered at 600 °C and 900 °C for 2 h and 6 h, as previously reported by Siwach et al.^57^. At 1300 °C, the concentration of perovskite started to decrease, while other phases, such as CoMn_2_O_4_, became apparent. The optimal sintering temperature was determined at 1100 °C when the perovskite phase deteriorates or transforms into a different phase at 1300 °C^58,59^. According to a report by Jonker, a solid-state technique for producing LaCo_x_Mn_1−x_O_3_ solid solutions was presented^60^. The process entails pre-firing La_2_O_3_, MnCO_3_, and CoCO_3_ powder mixtures for 15 h at 900 °C in air, then grinding the reaction mixture for 4 h in a ball mill. Finally, the pressed discs are fired once again. When comparing the current study to the work of Deniz Çoban Özkan et al.^61^, who synthesized LaMnO_3_ using the sol–gel method at temperatures of 500 and 850 °C for 2 h, it is observed that some irregularities persist. In the production of pure perovskite, crystalline impurities such as La_2_O_3_ or MnO_2_ are undesirable following annealing processes. Typically, elevating the annealing temperature results in the elimination of unwanted phases and promotes the growth of the perovskite structure.

The best temperature for crystallizing perovskite was determined to be 1100 °C, and it was utilized to make LaCo_0.3_Mn_0.7_O_3_ samples with different amounts of TiO_2_ or ZnO. These samples were then sintered at 1100 °C to investigate how adding TiO_2_ or ZnO affected their technological properties.

**Fig. 3:**
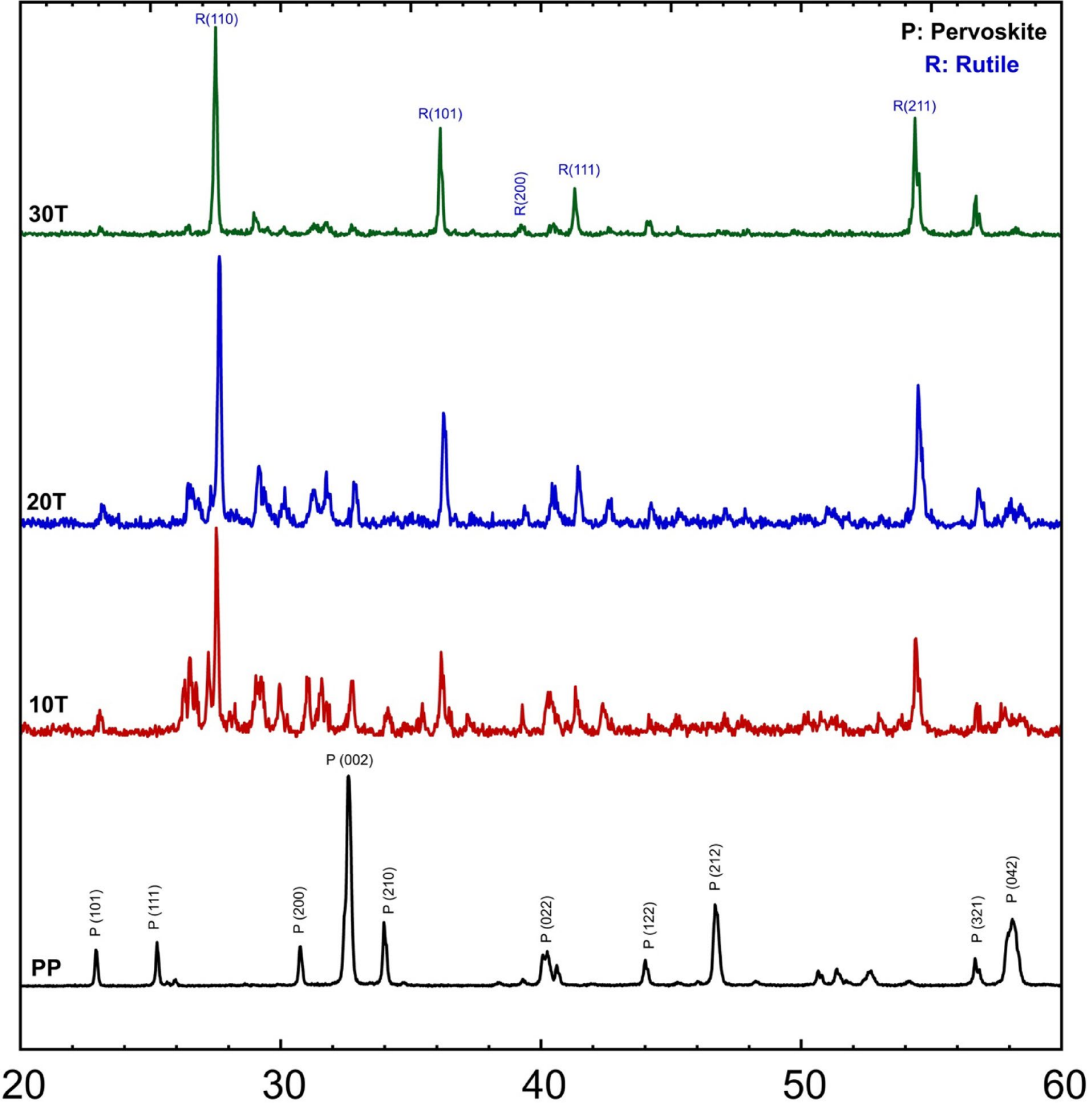
XRD patterns of TiO_2_/LaCo_0.3_Mn_0.7_O_3_ sintered bodies at 1100 °C.

The XRD patterns for PP, 10 T, 20 T, and 30 T sintered bodies, which contain LaCo_0.3_Mn_0.7_O_3_ and 10 wt%, 20 wt%, and 30 wt% of TiO_2_, respectively, are depicted in Fig. 3. The peaks observed at 27.54°, 36.24°, 39.20°, 41.33°, and 54.30° correspond to (110), (101), (200), (111), and (211) planes, respectively, of the rutile phase, as PDF#Card number 96-153-0151. The intensity of the rutile peaks increases at the expense of the perovskite peaks, which decrease gradually. It should be highlighted that the perovskite powder was synthesized by utilizing microwave-hydrothermal treatment. This synthesis method produces smaller crystallite sizes, leading to broader XRD peaks, which can be attributed to the size-broadening effect. This can lead to a reduction in peak intensities compared to Titania’s coarser particles.

As the concentration of TiO_2_ increased, a more significant decrease in perovskite concentration was noted until it was no longer present at a 30 wt% concentration of TiO_2_. This outcome can be attributed to the formation of metal–oxygen bonds in the form of MnO_6_ and TiO_6_. The TiO_2_ in these samples functions as an electron sink, facilitating the separation of electron–hole pairs generated in LaCo_0.3_Mn_0.7_O_3_.According to Thakur et al.^62^, the crystallization of TiO_2_ increases as the concentration of TiO_2_ particles rises, and this is accompanied by the formation of nanoparticles of LaCo_0.3_Mn_0.7_O_3_ covered with TiO_2_ particles. However, a decrease in the crystallization of perovskite is observed due to the interaction between the filler (TiO_2_) and the matrix, which results in an increase in the yield stress and decomposition of perovskite into a liquid phase as the concentration of TiO_2_ increases.

**Fig. 4:**
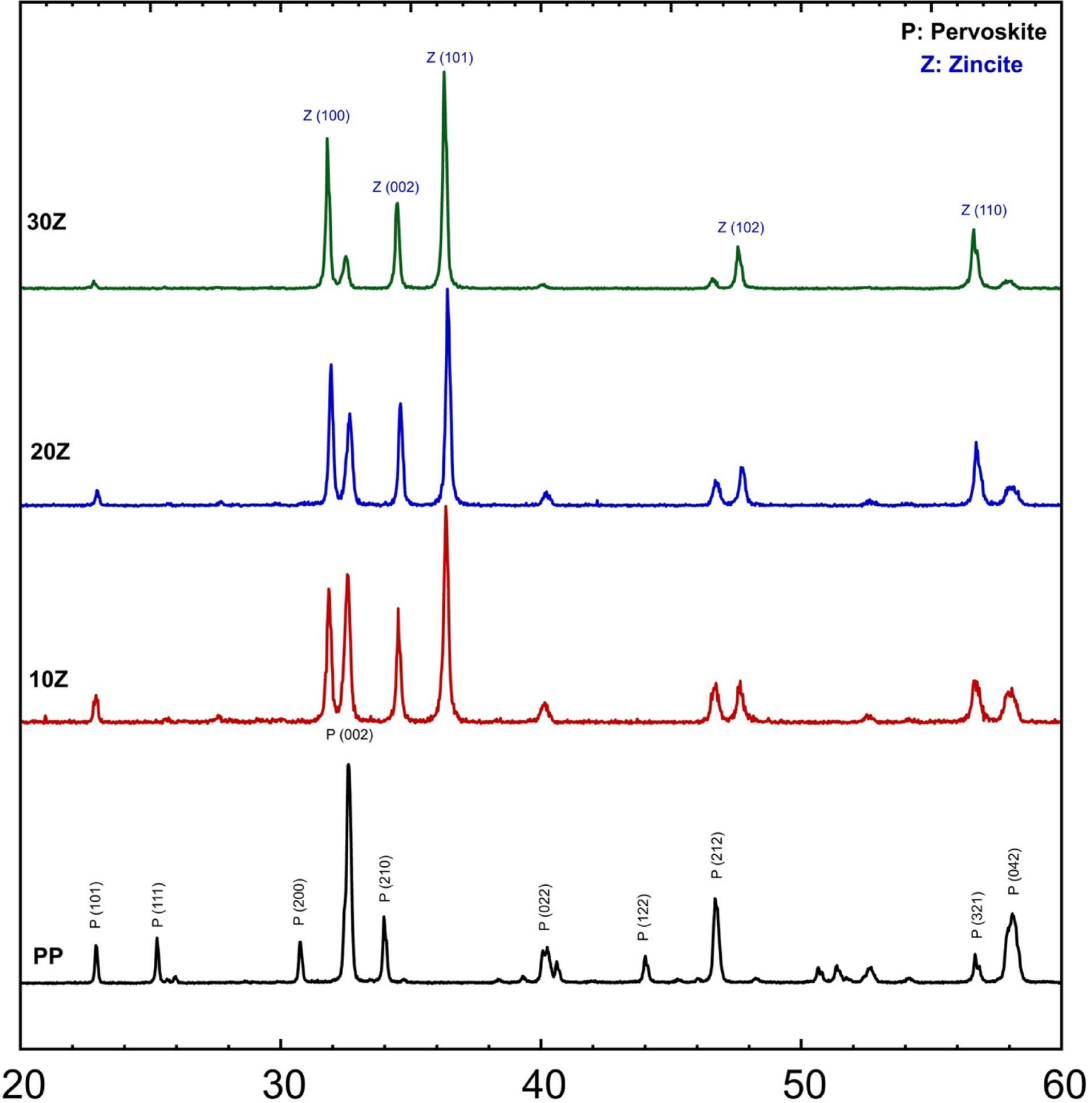
XRD patterns of ZnO/ LaCo_0.3_Mn_0.7_O_3_ sintered bodies at 1100 °C.

The X-ray diffraction (XRD) patterns for LaCo_0.3_Mn_0.7_O_3_ with varying ZnO concentrations are depicted in Fig. 4. These patterns show peaks at 31.79°, 34.57°, 36.33°, and 56.72°, which correspond to the (100), (002), (101), and (110) planes, respectively. These planes are characteristic of a hexagonal phase known as zincite, as reported in PDF#Card number 96-900-8878. It is important to note that the intensity of the zincite peaks increased while that of the perovskite peaks decreased gradually. This phenomenon has been attributed to the size-broadening effect, which can reduce peak intensities compared to larger particles of zinc oxide and titania. Nanosized perovskite powder is noteworthy because smaller crystallite sizes lead to broader XRD peaks, a well-known phenomenon.

The sizes of the crystallites in the perovskite powder samples were approximately 26 nm, 37 nm, and 39 nm, respectively, for calcination temperatures of 600, 1100, and 1300 °C. It is noteworthy that higher calcination temperatures resulted in larger crystallite sizes. Specifically, the crystallite sizes for the sintered bodies made from LaCo_0.3_Mn_0.7_O_3_with different concentrations of TiO_2_ were 42 nm, 44 nm, and 46 nm for samples T10, T20, and T30, respectively. Similarly, the crystallite sizes for the sintered bodies made from LaCo_0.3_Mn_0.7_O_3_with different concentrations of ZnO were 36 nm, 38 nm, and 49 nm for samples Z10, Z20, and Z30, respectively.

### SEM analysis

**Fig. 5:**
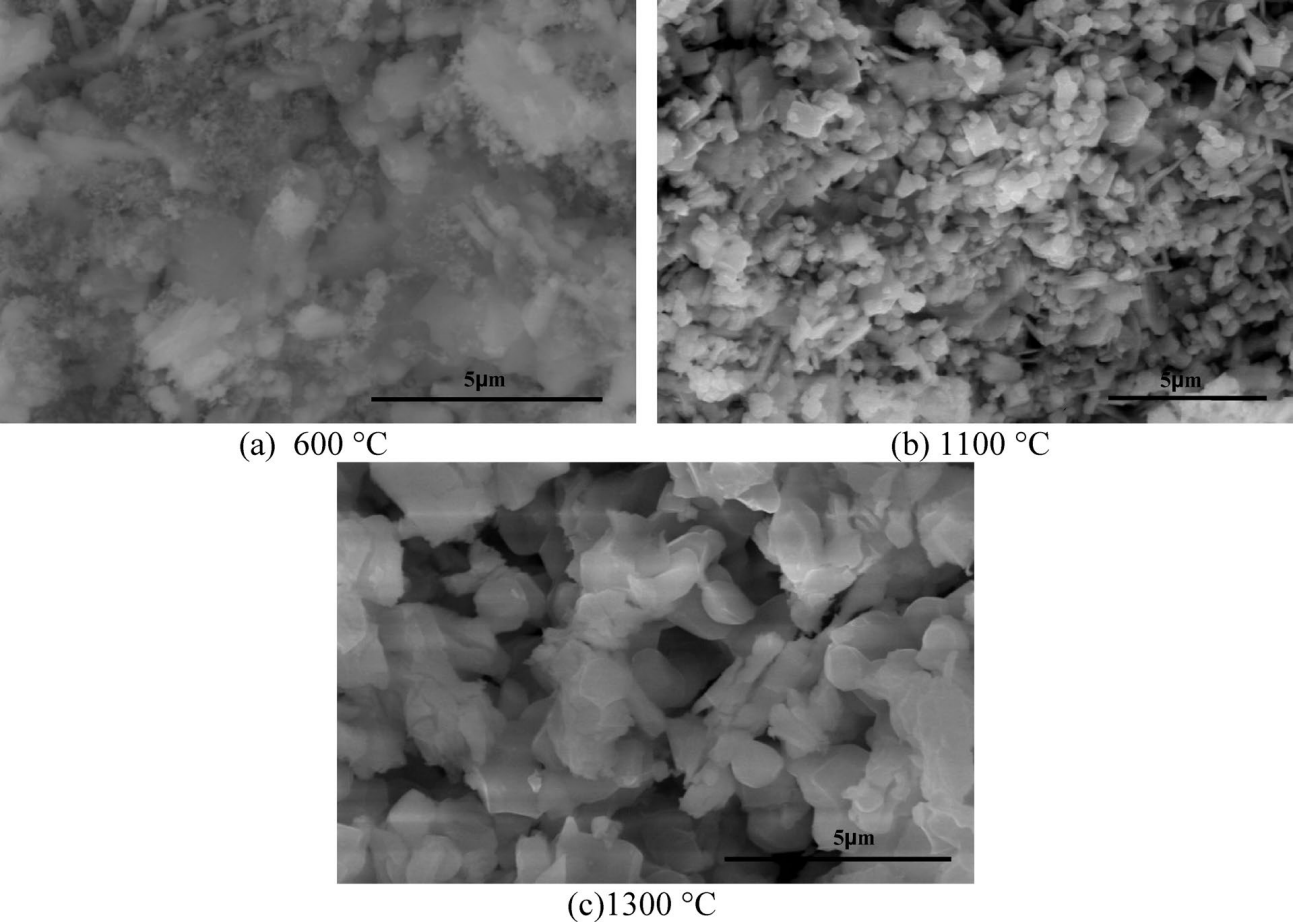
SEM images of LaCo_0.3_Mn_0.7_O_3_ powder calcined at different temperatures; (**a**) 600 °C, (**b**) 1100 °C, and (**c**) 1300 °C.

Figure 5 displays the SEM images of LaCo_0.3_Mn_0.7_O_3_powder (PP) calcined at 600 °C, 1100 °C, and 1300 °C. The sample PP calcined at 600 °C (Fig. 5a) shows agglomerated particles with an undefined structure, indicating that it is still amorphous, as confirmed by the XRD results. The sample PP calcined at 1100 °C (Fig. 5b) exhibits a well-defined structure, indicating good crystallinity with uniformly dispersed, regular grain sizes and a semi-spherical shape. After calcination at 1300 °C (Fig. 5c), an increase in grain size is observed, and a liquid phase is starting to develop. Consequently, it can be concluded that the surface morphology was significantly altered by the calcination temperature, as evidenced by the morphology of the microstructure, according to Ref^63^.

**Fig. 6:**
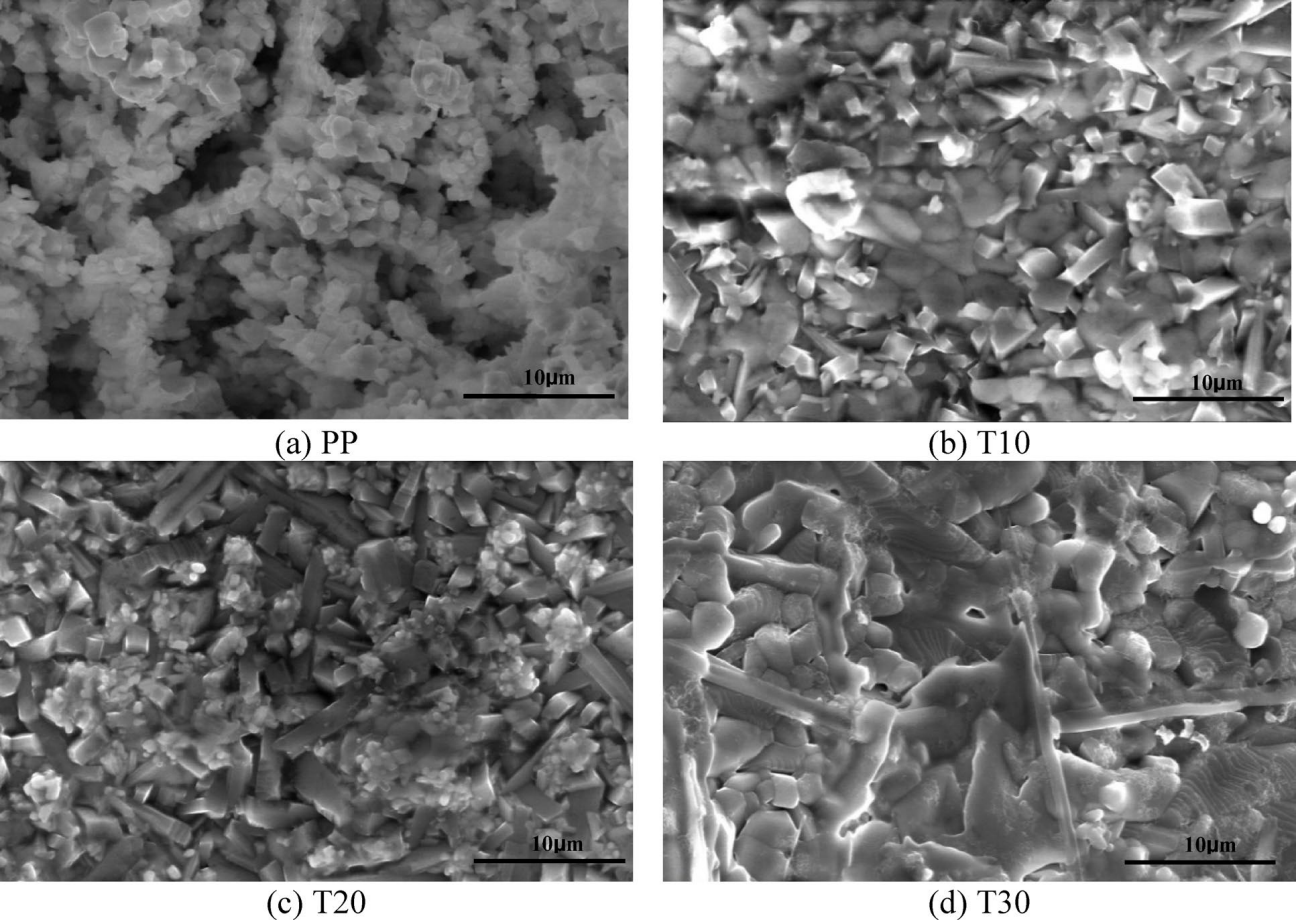
SEM images of LaCo_0.3_Mn_0.7_O_3_ and TiO_2_/ LaCo_0.3_Mn_0.7_O_3_ sintered bodies at 1100 °C, (**a**) PP, (**b**) T10, (**c**) T20, and (**d**) T30.

Figure 6 displays the SEM images of the PP, 10 T, 20 T, and 30 T bodies sintered at 1100 °C, which are made up of LaCo_0.3_Mn_0.7_O_3_ and 10 wt%, 20 wt%, and 30 wt% of TiO_2_, respectively. The SEM image of the PP body (Fig. 6a) shows semi-grains with considerable porosity. Perovskite appears cubic, whereas TiO_2_ appears in rod shapes. The rod-shaped concentration increases with increasing TiO_2_ content. Additionally, the grain growth of TiO_2_ increases with increasing TiO_2_, which inhibits the grain growth of the perovskite. The grain size of perovskite is smaller than that of the TiO_2_ particles. Moreover, a liquid phase is formed with increasing titania concentration compared to the PP sample. It was observed that with increasing TiO_2_, the pores decrease up to 20 wt% (Fig. 6c) of spherical TiO_2_ and then the porosity increases again when the TiO_2_ reaches 30 wt%. As seen in Fig. 6d, a more liquid phase is observed, which bulges^64^.

**Fig. 7:**
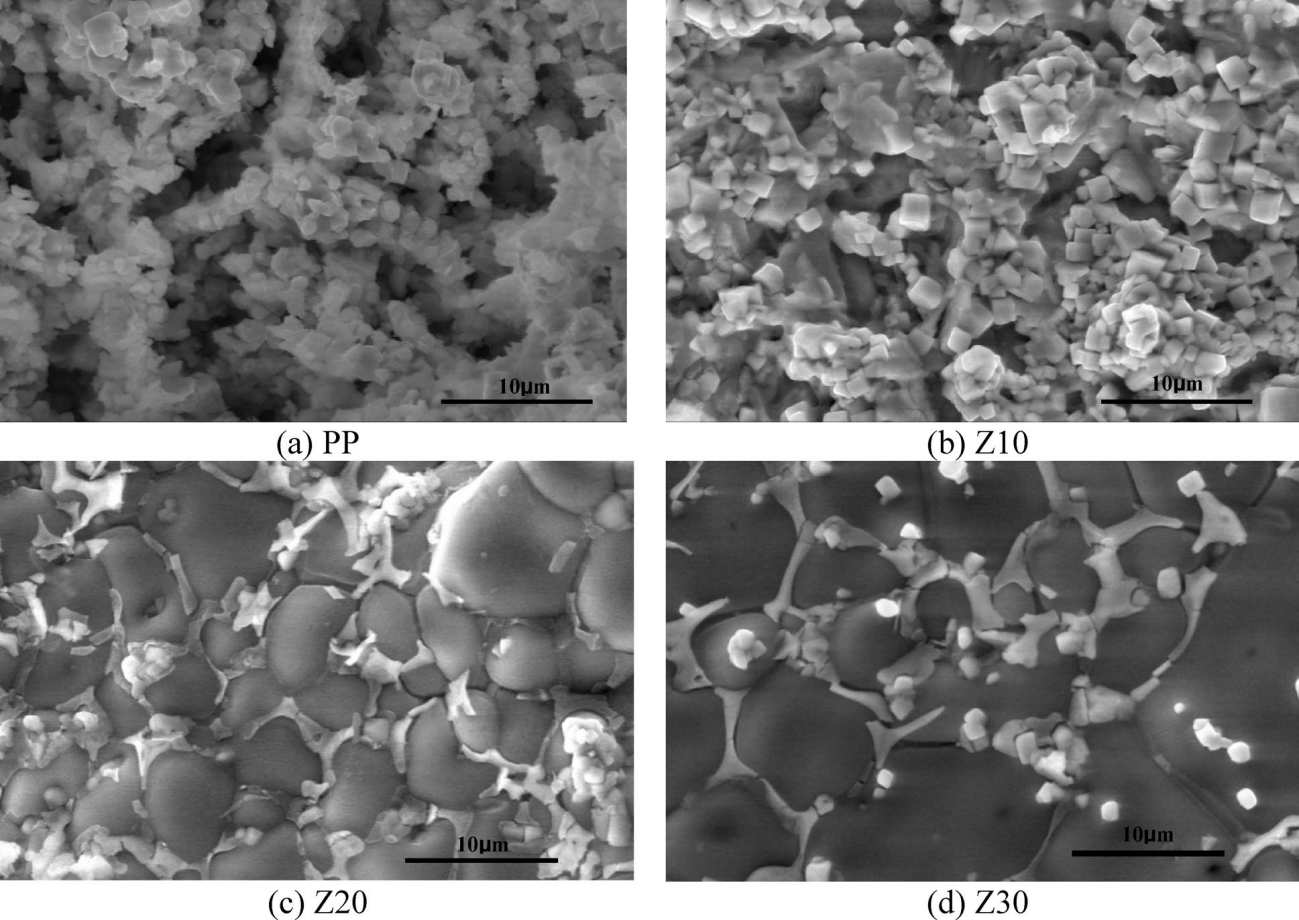
SEM images of LaCo_0.3_Mn_0.7_O_3_ and ZnO/ LaCo_0.3_Mn_0.7_O_3_ sintered bodies at 1100 °C, (**a**) PP, (**b**) Z10, (**c**) Z20, and (**d**) Z30.

Figure 7 displays Scanning Electron Microscope (SEM) images of the PP, 10Z, 20Z, and 30Z bodies sintered at 1100 °C. These bodies consist ofLaCo_0.3_Mn_0.7_O_3_ and 10 wt%, 20 wt%, and 30 wt% of ZnO, respectively. The SEM image of PP, which is LaCo_0.3_Mn_0.7_O_3_ sintered body, reveals semi-spherical grains with a notable level of porosity (Fig. 7a). In the 10Z sample, ZnO appears as a dark gray plated shape embedded between the perovskite, which has a cubic shape, as shown in Fig. 7b. With an increase in ZnO content, the size of ZnO increases and more compaction is observed, as depicted in Fig. 7c. The morphology of the cubic phase of the perovskite structure changes to a round-edge cubic form as the ZnO content increases, as evident in Fig. 7d. This may result in a larger contact surface between ZnO particles, promoting diffusion and density.

### XPS analysis

**Fig. 8:**
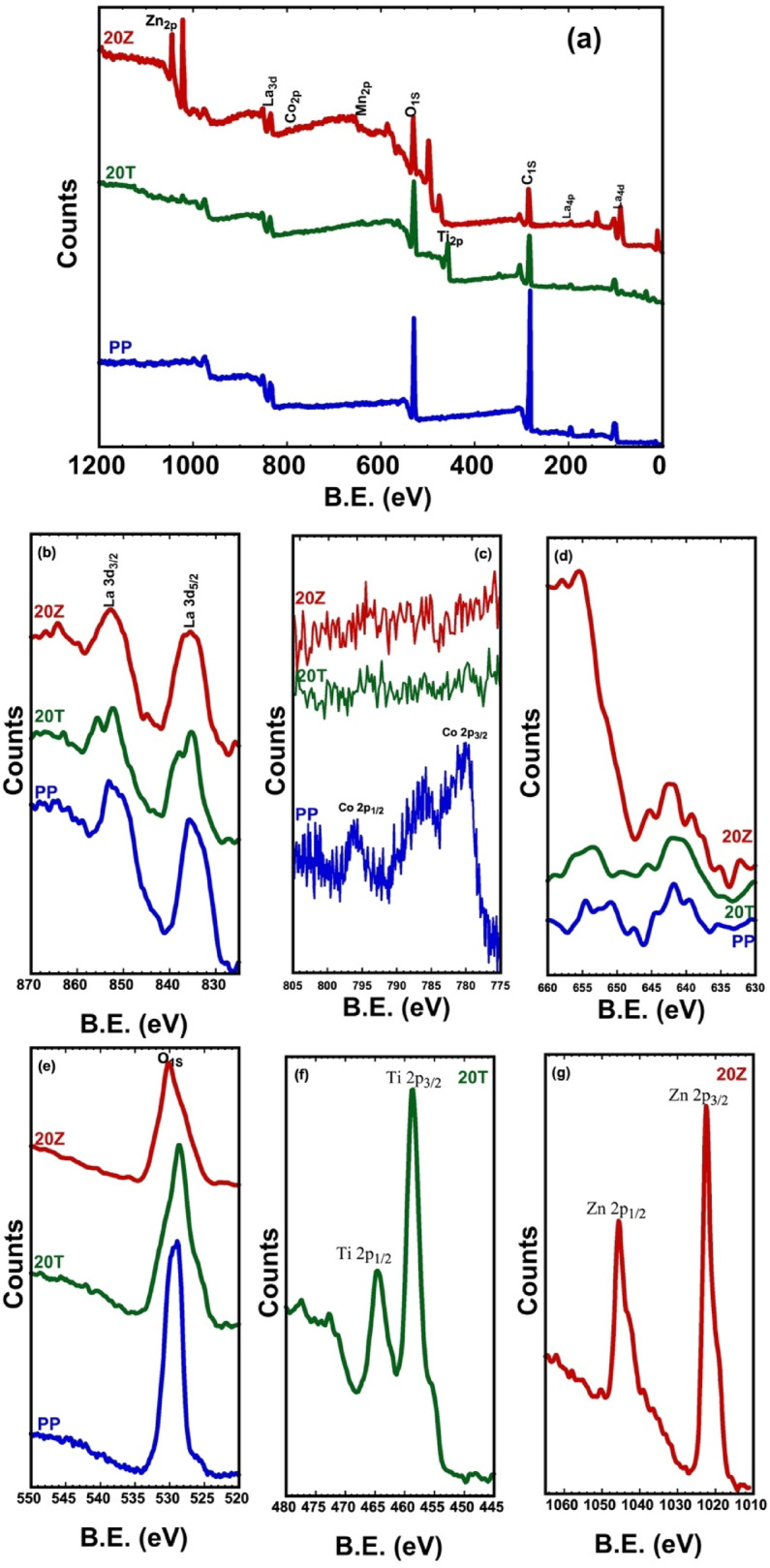
XPS spectra for LaCo_0.3_Mn_0.7_O_3_, 20 wt%TiO_2_/ LaCo_0.3_Mn_0.7_O_3_ and 20 wt%ZnO / LaCo_0.3_Mn_0.7_O_3_.

The elemental composition and oxidation states of components in lanthanum-cobalt-manganese pervoskite were investigated using X-ray photoelectron spectroscopy (XPS) on the calcined PP, 20 T, and 20Z samples. The XPS survey spectra of these three samples, depicted in Fig. 8a, revealed peaks associated with La 3d, Co2p, Mn2p, O1s, and C1s elements^65^.

Furthermore, Ti2p and Zn2p peaks were detected in the 20 T and 20Z samples, respectively. The high-resolution XPS spectra of La, shown in Fig. 8b, exhibited distinct spin–orbit components at approximately 835.8 eV for La3d_5/2_ and 853.2 eV for La3d_3/2_. The observed spin–orbit splitting value was about 17.4 eV, which is characteristic of the (+ 3) oxidation state of lanthanum ions^66^. Figure 8c displays the high-resolution XPS spectra of Co2p. The X-ray photoelectron spectroscopy (XPS) analysis indicates the existence of and peaks at 780.3 eV and 796.1 eV, respectively. The value for spin–orbit splitting was found to be around 15.8 eV. Moreover, two satellite peaks are visible at approximately 786.1 eV and 801.2 eV, which are typical of Co2p satellite peaks. Existing research suggests that determining the oxidation state of cobalt solely from binding energy is challenging. It is generally accepted that a spin–orbit splitting close to 15 eV can be associated with Co^3+^ species, while a splitting of about 16 eV is indicative of Co^2+^. This implies the presence of both (+ 2) and (+ 3) oxidation states in the Co ions^66^.

**Fig. 9:**
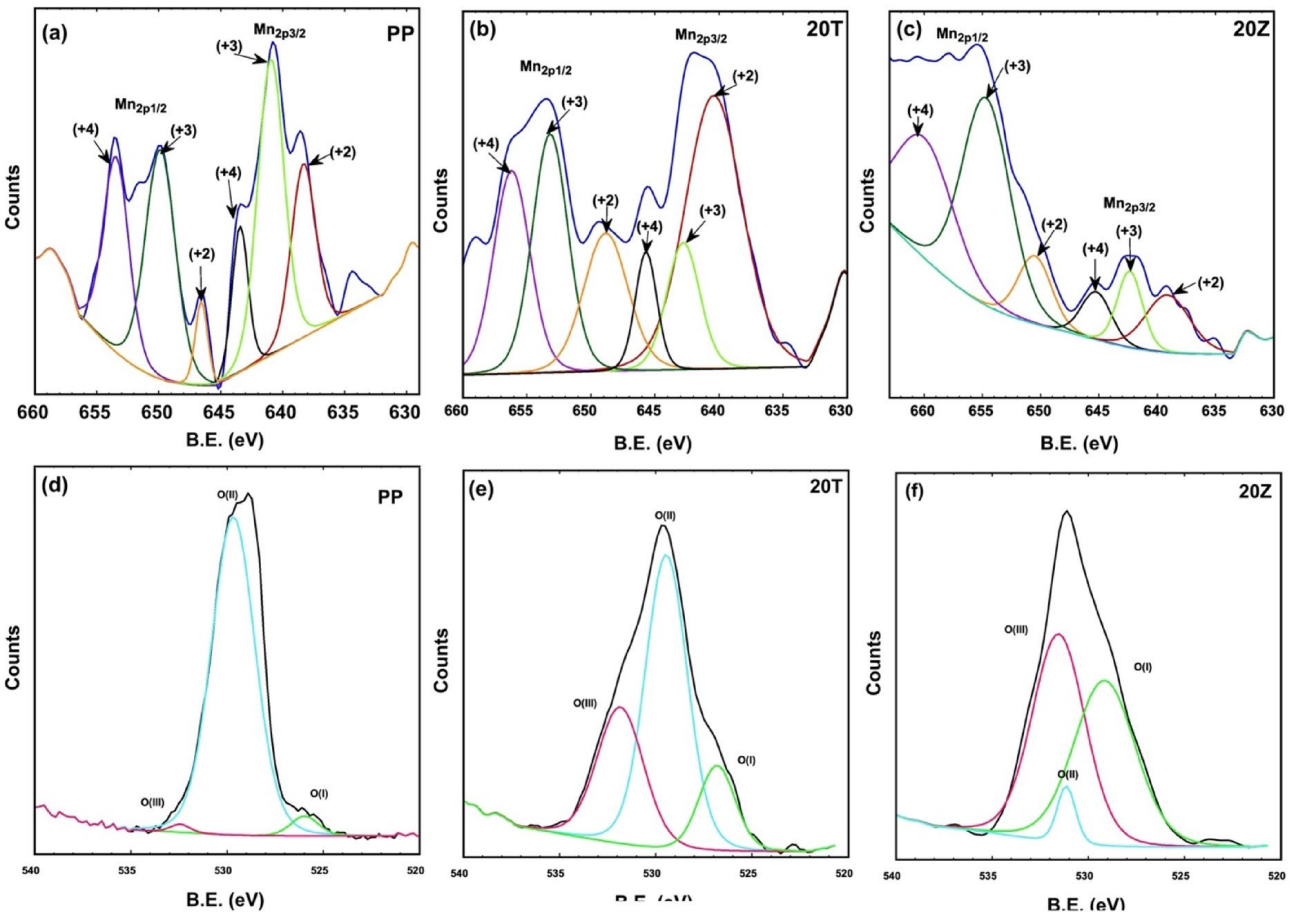
XPS spectra of O 1 s for LaCo_0.3_Mn_0.7_O_3_, 20 wt%TiO_2_/LaCo_0.3_Mn_0.7_O_3_ and 20 wt% ZnO/LaCo_0.3_Mn_0.7_O_3_.

The high-resolution XPS spectra of Mn2p, shown in Fig. 8d and deconvoluted in Fig. 9a–c reveal two prominent peaks centered around ~ 635–645 eV and ~ 647–660 eV, which are attributed to Mn2p_3/2_ and Mn2p_1/2_, respectively. The asymmetry of these peaks indicates the existence of multiple oxidation states. Deconvoluted XPS in Fig. 9a–c, shows peaks for oxidation states + 2, + 3, and + 4 and the atomic percentage is detailed in Table 1^66^. The high-resolution XPS spectrum of O1s, shown in Fig. 8e, reveals a peak around 528.8 eV, which is attributed to lattice oxygen (O^2–^) bound to metal ions in the crystal structure. An asymmetry in the O1s peak is observed for the 20 T and 20Z samples, suggesting the presence of oxygen vacancies (Vo) in oxygen-deficient regions. The deconvoluted XPS spectra of O1s for the samples are presented in Fig. 9d–f, where three oxygen species are used to fit the oxygen spectra. These include the lattice oxygen peak O(I) at lower binding energies, O(II) peaks representing oxygen species adsorbed in oxygen vacancies at intermediate binding energies, and the O(III) peak corresponding to physically adsorbed oxygen on the catalyst particle surface at higher binding energies. Compared to the pure perovskite, the modified perovskite samples exhibit a shift in oxygen species peak positions towards higher binding energies, particularly for the O(II) species. The O(II) species peaks were more prominent for Ti than for Zn. Table 1 provides a detailed breakdown of the surface oxygen species distribution data obtained through XPS analysis^67^.

**Table 1:** Detailed breakdown of the manganese and oxygen species distribution data obtained through XPS analysis.

Sample At%	Mn (+2)	Mn (+3)	Mn (+4)	O (I)	O (II)	O (III)
PP	20.5	54.1	25.4	9.25	83.27	7.48
20T	46	27	27	14.29	56.51	29.2
20Z	14	48	39	39.15	11.72	49.13

Figure 8f displays the high-resolution XPS spectrum of Ti 2p, which exhibits a Ti 2p_3/2_ peak at approximately 458.5 eV and a Ti 2p_3/2_ peak at roughly 465.7 eV. The separation between the Ti 2p_3/2_ and Ti 2p_1/2_ peaks, known as multiplet splitting, is typically about 7 eV, suggesting a (+ 4) oxidation state. The high-resolution XPS spectrum of Zn 2p is presented in Fig. 8g, showing a Zn 2p_3/2_ peak at around 1022.3 eV and a Zn 2p_3/2_ peak at about 1045.3 eV. The multiplet splitting between the Zn 2p3/2 and Zn 2p1/2 peaks was generally observed to be approximately 23 eV, indicative of (+ 2) oxidation.

### Physical properties

**Fig. 10:**
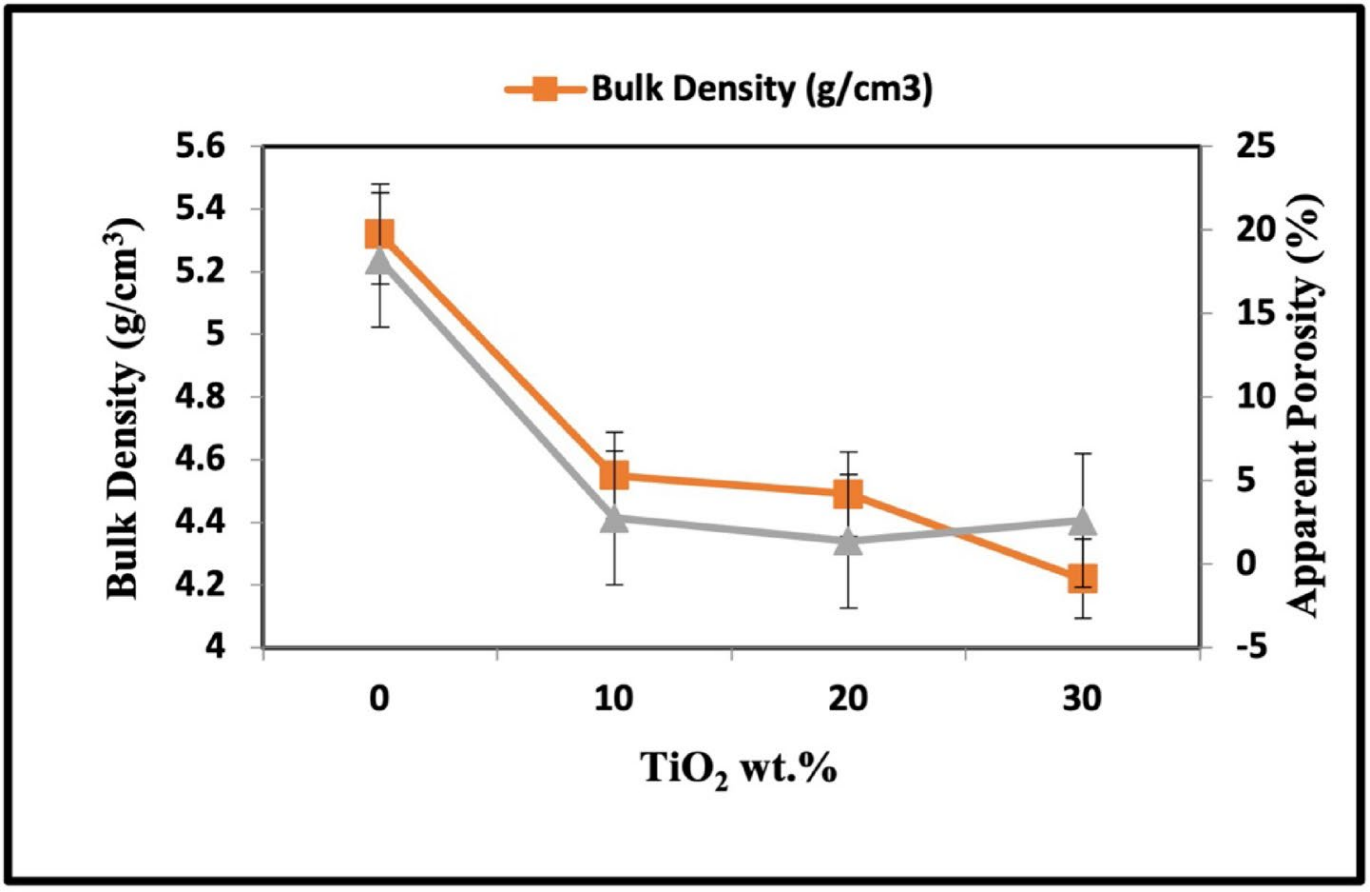
Bulk density and apparent porosity of TiO_2_/ LaCo_0.3_Mn_0.7_O_3_ sintered bodies at 1100 °C.

Figure 10 demonstrates the bulk density and apparent porosity alterations in response to the TiO_2_ content after sintering at 1100 °C. As the TiO_2_ content increases, the bulk density of the sintered samples decreases, primarily because the excessive addition of TiO_2_ did not enhance the sintering performance of the samples. The specific gravity of TiO_2_ is low (i.e., the weight of TiO_2_ per unit volume is less than that of the solid lanthanum manganite-lanthanum cobaltite solutions (LaCo_0.3_Mn_0.7_O_3_)^68,69^.The maximum density is observed for samples containing 10 wt% of TiO_2_ that reaches 4.55 g/cm^3^.The sintered specimens with no TiO_2_ had a maximum porosity of approximately 18%. When the amount of TiO_2_ added increased from 10 to 20 wt%, the apparent porosity of the samples decreased and reached 2.5%. Higher amounts of TiO_2_ cause an increase in system volume, resulting in a slight increase in porosity for the specimen with 30 wt%TiO_2_. Bulging was observed in the sample containing sufficient TiO_2_ due to forming a suitable amount of liquid phase with TiO_2_ addition that fills up the pores and activates the sintering process. The liquid wets the surfaces of the solid particles, reducing surface tension and improving particle contact, which helps in the rearrangement and consolidation of the samples. However, as the amount of TiO_2_ increases, a large amount of liquid phase is formed, causing bulging^70^.

**Fig. 11:**
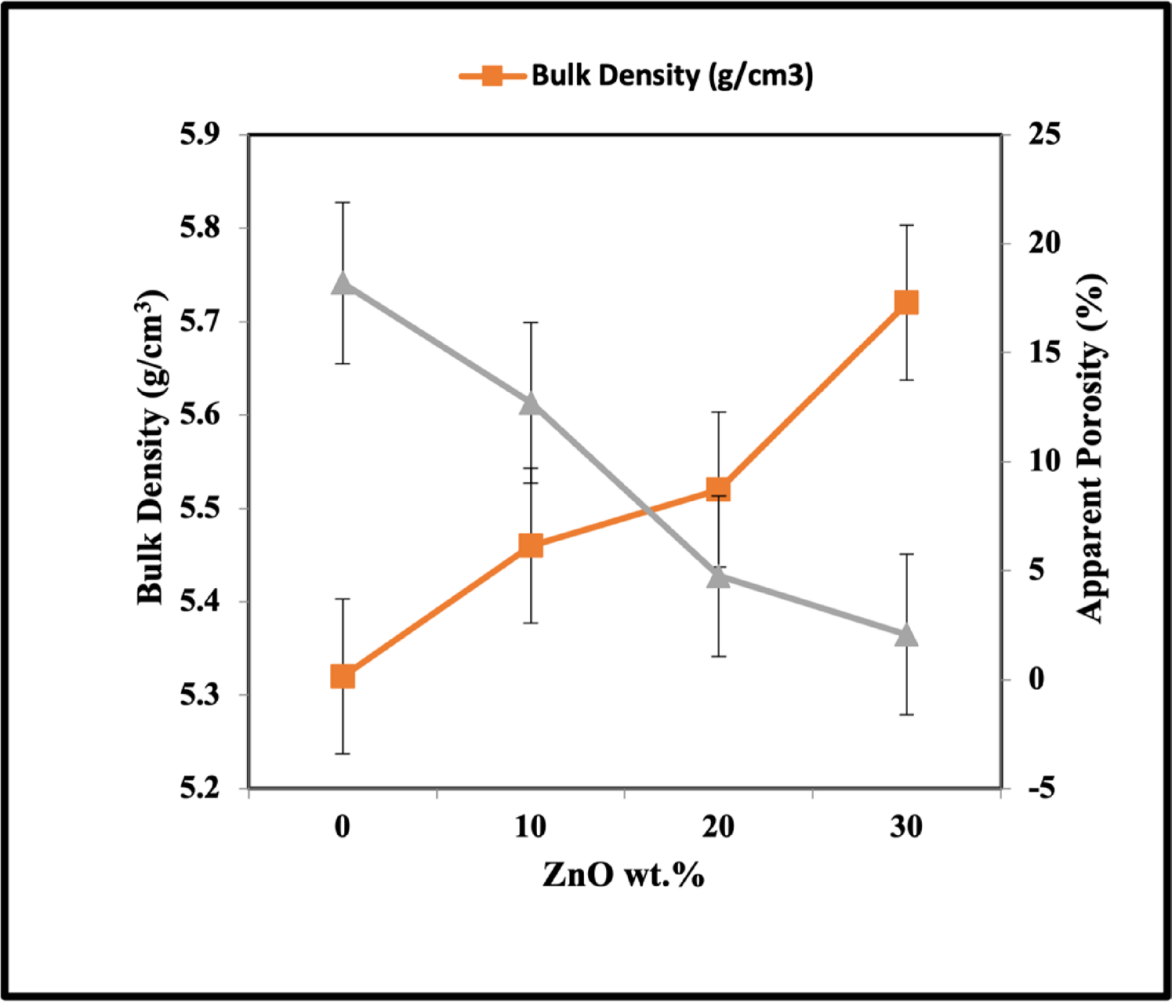
Bulk density and apparent porosity of ZnO/ LaCo_0.3_Mn_0.7_O_3_ sintered bodies at 1100 °C.

The sinterability characteristics of ZnO added to the perovskite structure are illustrated in Fig. 11. As the ZnO content increased from 10 to 30 wt%, the bulk density and the porosity values decreased. The maximum density and minimum porosity reach 5.7 and 2% after adding 30 wt% of ZnO, respectively. This behavior can be attributed to the sintering assist effect of the additive and the formation of a secondary phase that fills the pores between the grains^71,72^.

Some researchers suggest that Zn^2+^ ions might substitute at the A-site, resulting in cationic vacancies and altering the material’s characteristics^73,74^. Sintering is a process influenced by particle size distribution, dependent on nanosized ceramic powders’ high surface area and surface energy. This energy determines the sintering temperature, and reducing the powder size promotes better sintering conditions. During sintering, the nanoparticles within the material diffuse across particle boundaries, fusing the particles and forming a solid piece. However, a particle size that is too large or too narrow can negatively affect ceramic density. Thus, an optimal particle size distribution, including small and large particles, is necessary to achieve high green and sintering densities. This study used a mixture of nanosized perovskite particles and micro-sized ZnO particles to enhance sinterability^54–75^. The microwave hydrothermal technique demonstrates superior effectiveness in producing samples with enhanced sinterability at lower sintering temperatures (1100 °C) when compared to the method employed by Md. Mostafa Kamal et al. Their approach involved preparing LaMnO_3_ samples through a solid-state process at 1300 °C, resulting in materials with porosity ranging from 5.68 to 10.90% and density values between 3.7 and 4.3 g/cm^3^^76^.

### Vickers hardness

**Fig. 12:**
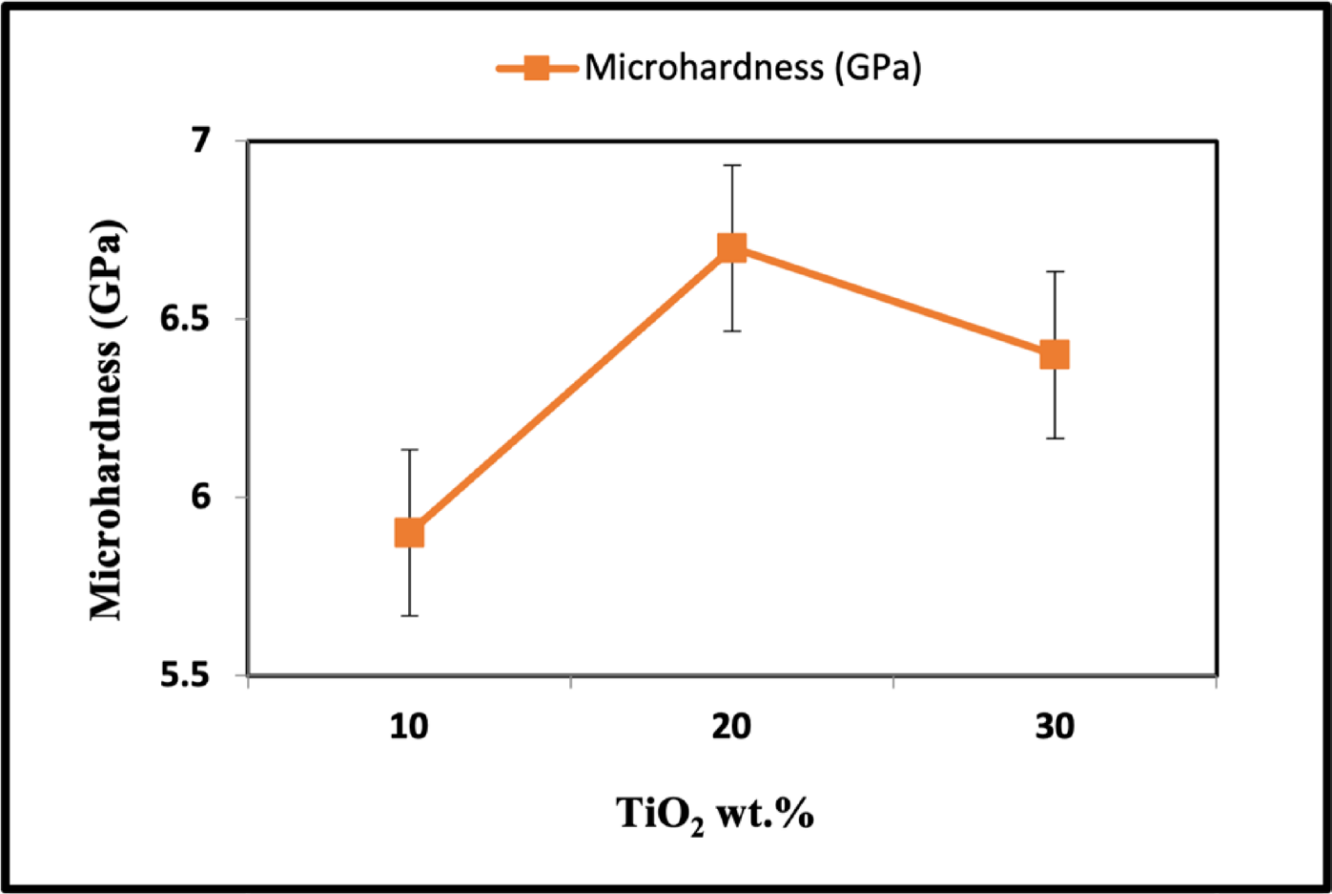
Hardness of TiO_2_/ LaCo_0.3_Mn_0.7_O_3_ sintered bodies at 1100 °C.

Vickers hardness of LaCo_0.3_Mn_0.7_O_3_ is very low, reaching 3.5 GPa due to the high porosity of these samples. The Vickers hardness results for the TiO_2_/LaCo_0.3_Mn_0.7_O_3_ ceramic samples with varying TiO_2_ percentages are shown in Fig. 12. Vickers hardness rose progressively from 5.8GPa (10 wt% TiO_2_) to 6.76GPa (20 wt% TiO_2_). Figures 10 and 11 illustrate that the Vickers hardness and porosity results are inversely related. The lowest porosity value matched the highest Vickers hardness value of 6.76GPa (20 wt% TiO_2_). A reduction in porosity was associated with the hardness, which increased as the TiO_2_ content increased to 20 wt%^77^. Nevertheless, with an additional increase of TiO_2_ to 30 wt%, the Vickers hardness decreased from 6.76 to 6.39 GPa. This is thought to be caused by the growth of TiO_2_ grains and an increase in the apparent porosity, which positively affects the hardness^78^, as shown in Fig. 10.

**Fig. 13:**
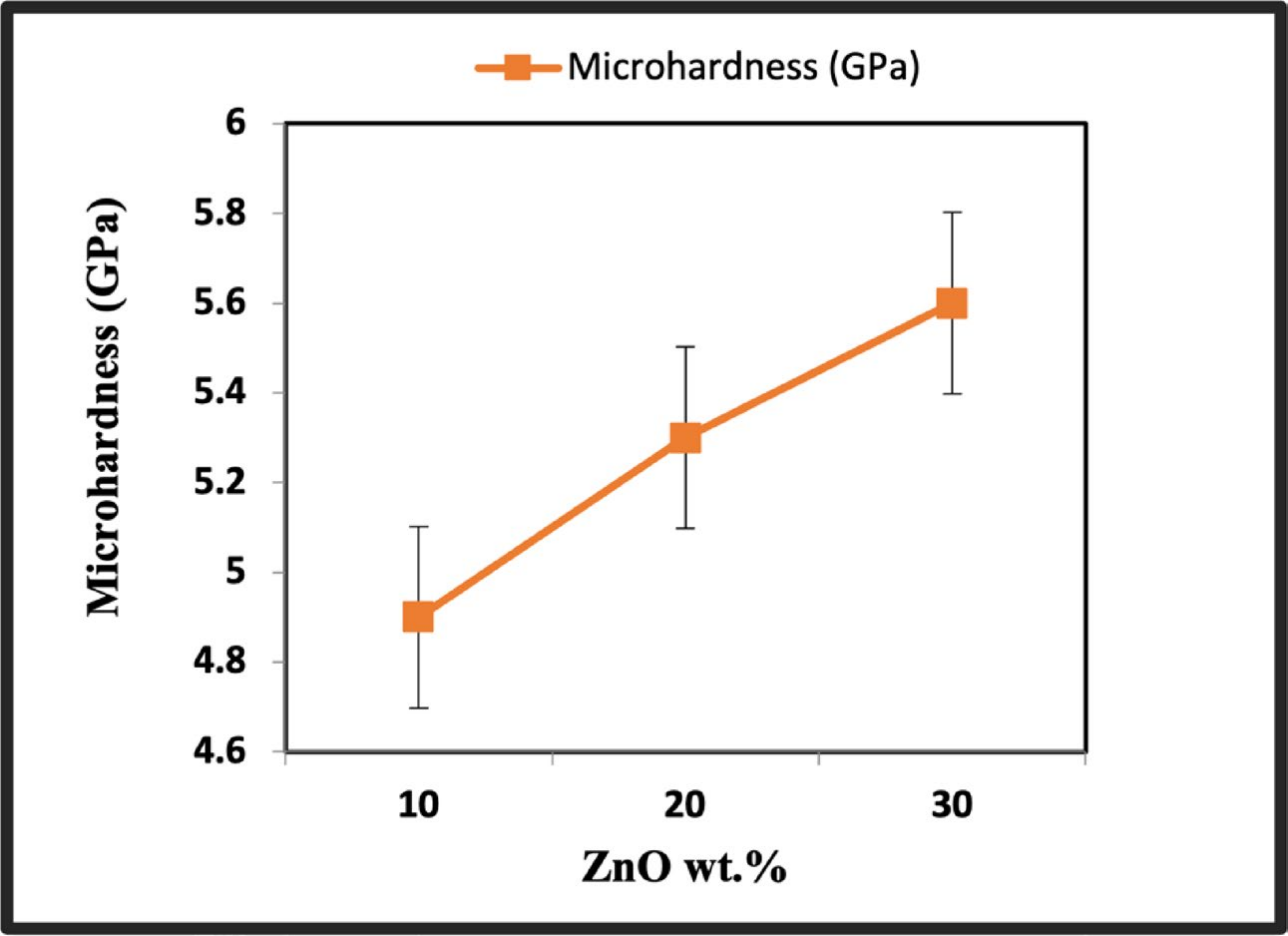
Hardness of ZnO/ LaCo_0.3_Mn_0.7_O_3_ sintered bodies at 1100 °C.

Figure 13 shows the relationship between adding ZnO on perovskite and Vickers hardness values. At low ZnO concentrations, the hardness value is 4.9 GPa compared to 4.2 GPa of sample PP. Increasing the ZnO content to 30 wt% rapidly increased the hardness value to 5.6 GPa. Grain size and density values were thought to play a role in the variations in hardness. Grain borders function as efficient barriers against dislocation pile-up in the neighboring grain and are called stress concentration locations^79^. Contrasting the current microwave hydrothermal synthesis method, Felipe Sanhueza et al*.* employed a rapid solution combustion technique to produce La_0.6_Sr_0.4_MnO_3_ perovskite. Their findings revealed that La_0.6_Sr_0.4_MnO_3_ achieved peak hardness values of approximately 0.4 GPa following calcination at 1500 °C^80^.

### Electrical conductivity and resistivity properties

**Fig. 14:**
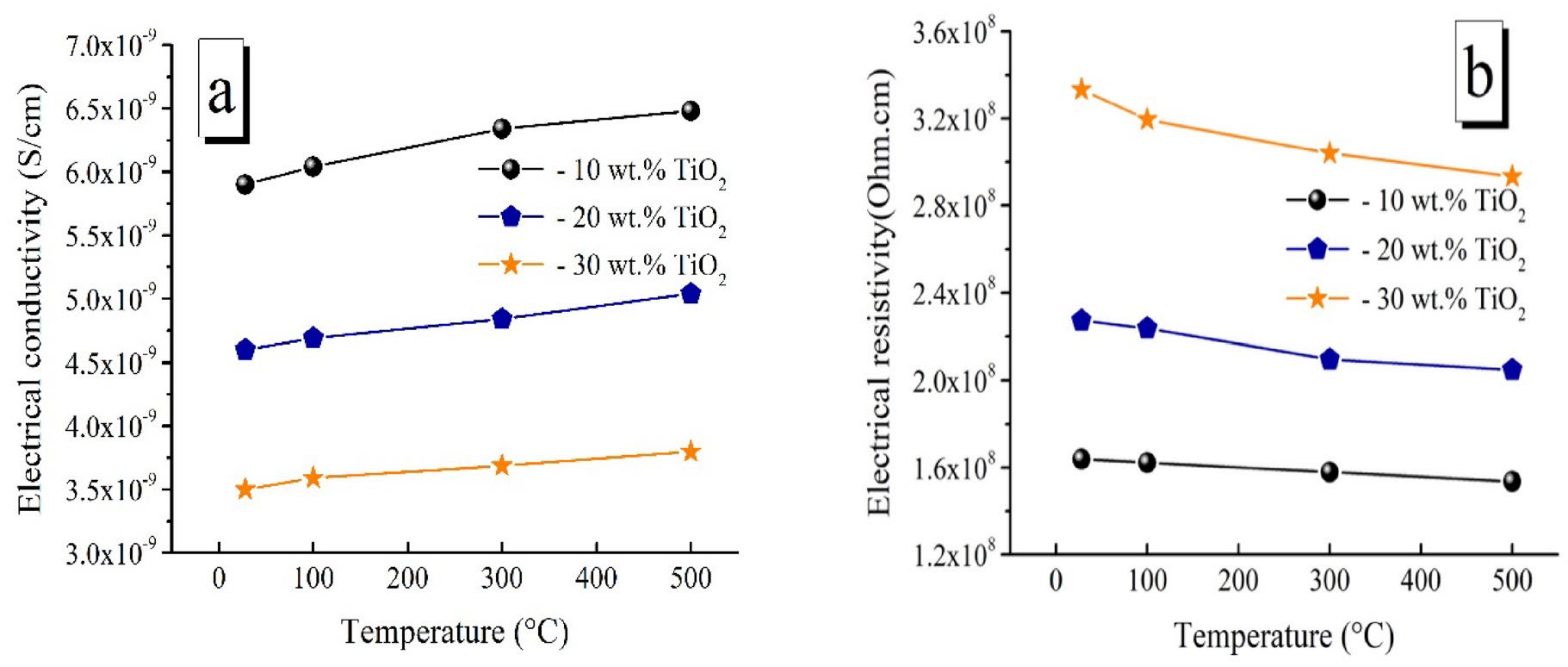
Electrical conductivity and Resistivity of TiO_2_/ LaCo_0.3_Mn_0.7_O_3_ sintered bodies at 1100 °C.

**Fig. 15:**
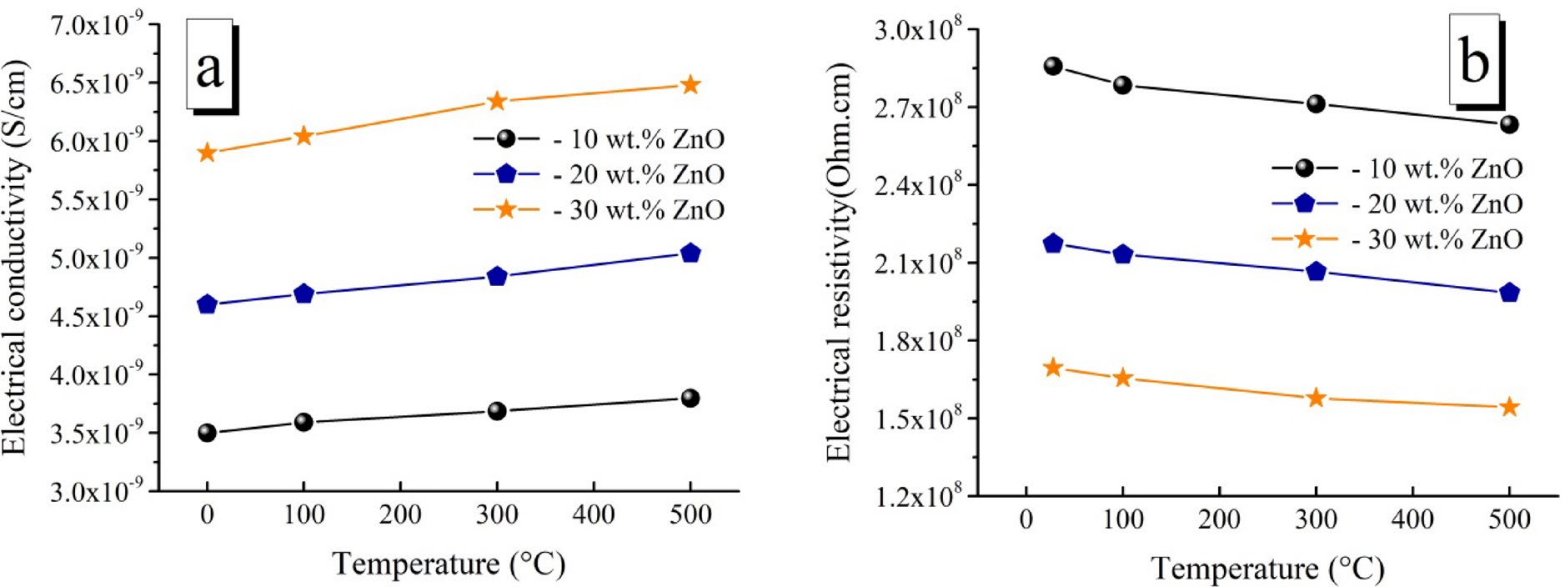
Electrical conductivity and Resistivity of ZnO/ LaCo_0.3_Mn_0.7_O_3_ sintered bodies at 1100 °C.

Conductivity and resistivity measurements were performed at 28° (room temperature), 100°, 300°, and 500 °C to examine the impact of the TiO_2_ and ZnO contents in the LaCo_0.3_Mn_0.7_O_3_ matrix, which was sintered at 1100 °C, on the electrical characteristics of the samples currently being researched. The significantly higher conductivity in LaCo_0.3_Mn_0.7_O_3_ does not happen in this work with higher porosity and fragility. It is observed that conductivity reaches to 2.5 × 10^–9^ S/cm and resistivity becomes 1.5 × 10^14^ Ω cm. The conductivity and resistivity of each oxide sample are shown in Figs. 14 and 15, respectively. For all measuring temperature, when the TiO_2_ content in the samples was increased from 10 to 30 wt%, resistivity increase and a decrease in conductivity were observed, as shown in Fig. 14. Generally, when we increase the measurement temperature, we observe a positive impact on electrical conductivity and a negative impact on electrical resistance. This could be attributed to the fact that electrical conductivity in ceramic insulators increases slightly with temperature while resistivity decreases due to thermal energy, which may attract electrons from the valence band to the conduction band, even if insulators have a wide band gap. These results are in-line with those reported in Refs.^81,82^.

The findings revealed that while TiO_2_ does not react with perovskite to change its crystal structure, it causes significant stress on the material, causing it to reduce the grain size and transition into a liquid phase. TiO_2_ is primarily separated at the perovskite grain borders and the grain surface, which coexists with the matrix. Thus, the surface disorder layer of the perovskite granules is caused by TiO_2_. Because of its superior insulating qualities, segregated TiO_2_ creates a barrier to charge transport, increasing resistivity^83,84^.

On the other hand, at all measuring temperatures, with an increase in ZnO content, as seen in Fig. 15, the conductivity increases, and the resistance decreases for all samples. When a sample has a high concentration of LaCo_0.3_Mn_0.7_O_3_, oxygen vacancies cause pairs of Mn^+2^ and Mn^+3^ ions to form, which supports the Zener^85,86^. Double-exchange mechanism and causes leakage into the lattice^85,87–89^. This causes lattice leakage/loss in the electrical nature. This implies that conducting pathways or leakage/loss in the lattice of composites is reduced from high to low ZnO concentration, resulting in increased conductivity. This is explained by free charge carriers in the lattice, which are released because of oxygen vacancy defects^84–86^. With ZnO, free charge carriers in Mn^+2^ ions were produced by oxygen vacancies in the LaCo_0.3_Mn_0.7_O_3_ lattice, resulting in more free charge carriers (per unit cell). Consequently, it is reasonable to anticipate higher conduction/conductivity in lattices with higher ZnO content than those with low ZnO content.

Other works^90–92^ showed that an increase in conduction with increased ZnO contents due to the presence of extra ZnO and the eutectic product among La_2_O_3_, ZnO, and Mn_2_o_3_ at the boundary of perovskite, thus impeding proton conduction. In addition, when ZnO was added to LaCo_0.3_Mn_0.7_O_3_, A-site-deficient LaCo_0.3_Mn_0.7_O_3_ and some Mn_2_O_3_ may have formed, leading to a change in the bulk composition. This compositional change caused a slight decrease in bulk conductance. Another study^93^ said that the ionic radius of La^3+^ and Zn^2+^ differ significantly, which are 1.06 Å and 0.74 Å, respectively, making it difficult for a high amount of La^3+^to enter the ZnO lattice and replace Zn^2+^. La mainly exists at the ZnO grain boundary as a La-rich phase. A part of La^3+^ will enter the ZnO lattice, leading to a less solid solution La formed in ZnO grains.So, the vacancy compensation of trivalent metal cation will occur, which increases the grain resistivity. Meanwhile, the La-rich phase distributed in the grain boundary hindered the conductive path between the ZnO grains.

### Magnetic properties

**Fig. 16:**
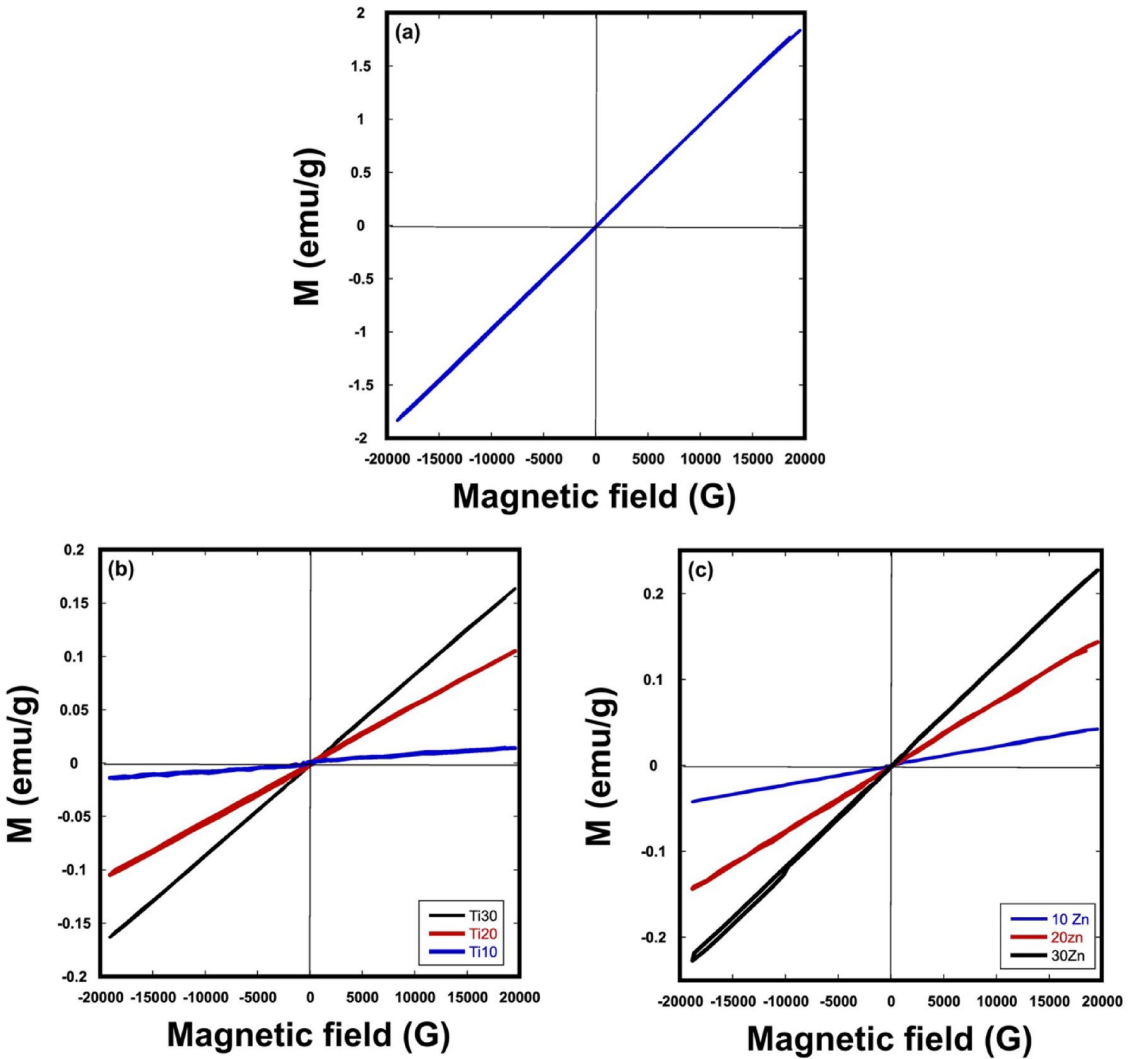
Magnetic properties of (**a**) LaCo_0.3_Mn_0.7_O_3_, (**b**) TiO_2_/ LaCo_0.3_Mn_0.7_O_3_ and (**c**) ZnO/LaCo_0.3_Mn_0.7_O_3_ sintered bodies at 1100 °C.

Figure 16 shows the magnetic characters of samples sintered at 1100 °C. Every sample had paramagnetic characteristics, as can be shown. In general, the low magnetic value of LaCo_0.3_Mn_0.7_O_3_, was observed after incorporation of cobalt according to Feriel Zdiri et al. This may be related to the disordered state induced by Co in the lattice in the parent compound due to the different sizes of cations in B site^94–98^. The addition of ZnO or TiO_2_ has been shown to decrease paramagnetic behavior. The nonmagnetic behavior of titanium ions in the event of TiO_2_ addition is the cause of the decrease in magnetic character. Neel’s theory and the super-exchange interaction mechanism^94–96^ explain this. Ti^4+^ ions strongly prefer the B site; therefore, they occupy it by dispensing with B ions, which reduces the strength of the exchange contact between the A and B sites. As a result, the saturation magnetization decreases with increased TiO_2_ content^94–96^. Zinc oxide (ZnO) is a diamagnetic material; however, it may be magnetically useful by doping it with magnetic elements or joining it with magnetic materials to form a core–shell structure. Hence, increasing the ZnO content led to a decrease in the magnetic characteristics of the samples^99^. Doping LaMnO_3_ with Co and introducing TiO_2_ or ZnO improves the magnetic characteristics compared to La_0.7_Sr_0.3_MnO_3_^100^ where magnetization increased in the opposite direction of the externally applied magnetic field (anti-S shaped) when the field strength exceeded ± 1500 G. This demonstrates the inherent diamagnetic behavior of La_0.7_Sr_0.3_MnO_3_ produced through co-precipitation and sol–gel auto-combustion methods. This phenomenon can be attributed to the presence of potential point defects, such as vacancies, which may form on or near the surface of the polycrystalline sample during the synthesis process.

## Conclusion

In conclusion, this study examined the effects of TiO_2_ and ZnO additions on the structural, physical, electrical, and magnetic properties of LaCo_0.3_Mn_0.7_O_3_ perovskite ceramics which was successfully prepared by a low-temperature microwave hydrothermal method after being treated at 1100 °C. XRD analysis revealed the formation of rutile TiO_2_ and hexagonal ZnO phases with increasing additive content. The crystallite sizes ranged from 36–49 nm for the sintered bodies. SEM imaging showed changes in microstructure, with TiO2 appearing as rod-shaped particles and ZnO as plate-like grains embedded between perovskite particles. XPS confirmed the presence of multiple oxidation states for Mn (+ 2, + 3, + 4) and Co (+ 2, + 3). The bulk density reached a maximum of 4.55 g/cm^3^ with 10 wt% TiO_2_ addition and 5.7 g/cm^3^ with 30 wt% ZnO. Porosity decreased to a minimum of 2.5% for 20 wt% TiO_2_ and 2% for 30 wt% ZnO. Vickers hardness increased to 6.76 GPa with 20 wt% TiO_2_ and 5.6 GPa with 30 wt% ZnO. Electrical conductivity decreased with TiO_2_ content but increased with ZnO content, reaching 2.5 × 10^–9^ S/cm for pure LaCo_0.3_Mn_0.7_O_3_. All samples exhibited paramagnetic behavior, with magnetization decreasing upon TiO_2_ or ZnO addition. These results demonstrate the ability to tailor the properties of LaCo_0.3_Mn_0.7_O_3_ ceramics through controlled addition of TiO_2_ and ZnO for potential applications in electronic and magnetic devices.

## Data Availability

All data generated or analyzed during this study are included in this published article.
